# Early Life Factors Associated with Lean Body Mass in Spanish Children: CALINA Study

**DOI:** 10.3390/children9050585

**Published:** 2022-04-20

**Authors:** Diana Paola Córdoba-Rodríguez, Iris Iglesia, Alejandro Gómez-Bruton, María Luisa Álvarez Sauras, María L. Miguel-Berges, Paloma Flores-Barrantes, José Antonio Casajús, Luis A. Moreno, Gerardo Rodríguez

**Affiliations:** 1 Departamento de Nutrición y Bioquímica, Facultad de Ciencias, Pontificia Universidad Javeriana, Bogotá 110231, Colombia; d.cordoba@javeriana.edu.co; 2 Growth, Exercise, Nutrition and Development (GENUD) Research Group, Instituto Agroalimentario de Aragón (IA2), Universidad de Zaragoza, 50009 Zaragoza, Spain; bruton@unizar.es (A.G.-B.); mlmiguel@unizar.es (M.L.M.-B.); pfloba@unizar.es (P.F.-B.); joseant@unizar.es (J.A.C.); lmoreno@unizar.es (L.A.M.); gerard@unizar.es (G.R.); 3 Instituto de Investigación Sanitaria Aragón (IIS Aragón), 50009 Zaragoza, Spain; mlalvarez@iisaragon.es; 4 Red de Salud Materno Infantil y del Desarrollo (SAMID), Instituto de Salud Carlos III, 28029 Madrid, Spain; 5 Primary Care Interventions to Prevent Maternal and Child Chronic Diseases of Perinatal and Developmental Origin Network (RICORS), RD21/0012/0012, Instituto de Salud Carlos III, 28029 Madrid, Spain; 6 Departamento de Fisiatría y Enfermería, Facultad de Ciencias de la Salud y del Deporte (FCSD), Universidad de Zaragoza, 50009 Zaragoza, Spain; 7 Centro de Investigación Biomédica en Red de Fisiopatología de la Obesidad y Nutrición (CIBERObn), Instituto de Salud Carlos III Madrid, 28029 Madrid, Spain; 8 Área de Pediatría, Universidad de Zaragoza, 50009 Zaragoza, Spain

**Keywords:** lean body mass, muscle cross-sectional area, perinatal factors, schoolchildren

## Abstract

Early life is critical for the programming of body composition. The literature links perinatal factors with fat mass development and its future effects (e.g., obesity); however, little evidence exists between early life factors and lean body mass (LBM). This study follows up on a cohort of 416 Spanish children at ages six to eight, previously evaluated at birth in the CALINA study. Here, we studied the association between early life factors, LBM, and limb strength. Parental origin/nutritional status, maternal smoking during pregnancy, gestational diabetes/weight gain/age, birth weight (BW), early feeding, and rapid weight gain (RWG) were collected from primary care records. Bioimpedance analysis, dual-energy X-ray absorptiometry, peripheral quantitative computed tomography, and a handgrip/standing long jump test were used to assess fat-free mass index (FFMI), total lean soft tissue mass index (TLSTMI), muscle cross-sectional area index (MCSAI), and limb strength, respectively. In girls, maternal smoking, gestational age, and BW were positively associated with FFM/LSTM. In boys, the parents’ BMI, BW, and RWG were positively associated with FFM/LSTM. BW was associated with handgrip strength in both. Maternal BMI in girls and RWG in boys were negatively associated with the standing long jump. Early life programming plays a key role in determining LBM in children.

## 1. Introduction

Lean body mass (LBM) includes skeletal muscle mass, nonfat components of internal organs, and extracellular fluid [1]. Skeletal muscle mass is the main component of LBM and is involved in metabolically active processes such as regulating resting energy expenditure, glucose uptake, and myokines secretion. Additionally, it is the main reservoir of amino acids to maintain protein synthesis and a determinant for posture, locomotion, and bone health [2].

LBM has been considered essential in maintaining growth, normal development, and systemic glucose metabolism in children [3]. High LBM is associated with a reduced risk of cardiovascular disease [4], and improvements in bone health (bone mineral density and structure in both sexes during childhood) [5,6] and cognitive development [7]. Studies have shown that children and adolescents with low LBM have a higher cardiometabolic risk [4,8,9,10,11].

Some environmental and nutritional factors during the prenatal period and early postnatal development (fetal programming) [12] were reported to affect the growth and development of muscle mass/lean mass in the long term, even producing permanent effects (e.g., less lean mass [13] and less grip strength [14]). These factors include parental nutritional status, gestational diabetes mellitus [15], intrauterine growth restrictions, early nutritional status [16], breastfeeding, and rapid weight gain (RWG) [17]. Parental nutrition status as represented by body mass index (BMI) has been shown to be associated with the lean mass index (LMI) of children between 5 and 21 years of age [15]. Maternal hyperglycemia in pregnancy was associated with low LBM, both in early childhood [18] and in adolescence [19].

Recent studies have shown that children born small for their gestational age have less lean mass at birth and less muscle growth between two months and eight years of age [20] than those born with adequate weight [21]. Conversely, infants born large for their gestational age have higher lean mass values in later life [22]. A study in Spanish adolescents [23] showed that fat-free mass (FFM) was significantly associated with birth weight (BW) in girls, independent of pubertal stage, age, socioeconomic status, gestational age, physical activity, and height. Low BW is often associated with RWG (catch-up), characterized by the accelerated growth of adipose tissue outpacing lean mass growth [24], an association that has been observed even in adulthood [25]. Likewise, a systematic review and meta-analysis comparing the effect of breastfeeding and formula feeding on the body composition of premature infants found that formula-fed infants had a lower FFM than their breastfed counterparts at 32 weeks, corrected for gestational age [17]. On the other hand, full-term formula-fed babies have been found to have a higher FFM during the first year of life [26].

These environmental and nutritional factors, present during the prenatal and early postnatal period, tend to cluster in social groups, potentially confounding the results. Studies have shown that low socioeconomic status was associated with less muscle mass, and this may be attributed to mothers with low educational levels being more likely to smoke during pregnancy [27].

The study of early life factors can help build scientific evidence concerning the long-term consequences of prenatal and postnatal influences on the rearing and development of LBM/FFM and the key role it may play in the “programming” of chronic diseases. In addition, it could reinforce the importance of optimizing maternal and child health during the “first 1000 days” [28].

Given the extensive literature related to early life factors and the development of fat mass and its future effects (obesity, among others) [29,30,31] and the limited existing evidence on the link between early life factors and LBM and the impact on its function (strength) in later childhood, this study aims to explore the association between perinatal factors and LBM and limb strength in a Spanish cohort of children.

## 2. Materials and Methods

### 2.1. Study Participants

**Figure 1:**
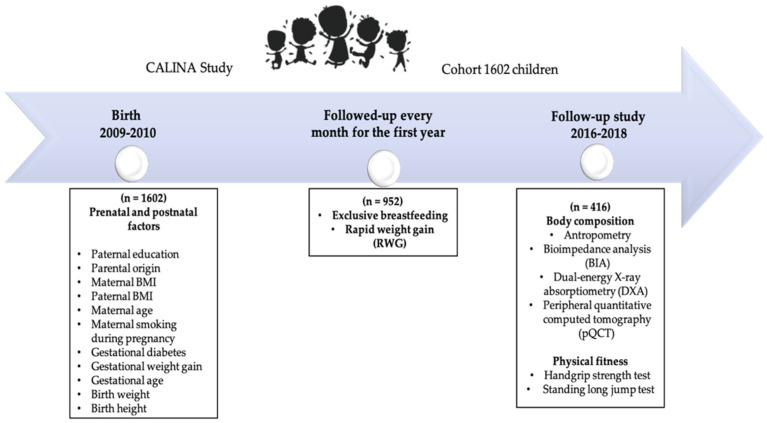
Diagram of initial recruitment and subsequent follow-up examinations of the CALINA cohort.

The participants in this study are part of the CALINA (Growth and feeding during infancy and early childhood in children of Aragon) longitudinal observational study involving a representative cohort of children born in Aragon (Spain) between 2009 and 2010. The first sample included 1602 newborns [32], followed-up monthly for the first year and then yearly until the ages of six to eight. From 2016 to 2017, the families initially recruited in Zaragoza-Aragon (*n* = 952) from the baseline examination were invited to participate in this follow-up study. Four hundred and sixteen of the 952 families agreed to their children’s participation in this body composition evaluation in the laboratory of the University of Zaragoza when the children were between the ages of six and eight. The measures and timepoints collected in these analyses are presented in Figure 1.

In compliance with ethical requirements, the study was carried out following the Declaration of Helsinki 1964 (revised Fortaleza 2013) and approved by the Ethics Committee in Clinical Research of the Government of Aragon (ref. PI ICS108/0088, Spain) on 4 June 2018. In 2013, the same Committee approved the follow-up referred to in this manuscript (11/09/2013. Ref. CPPI13/00105, Spain).

The parents provided written informed consent for their children’s participation in this evaluation. The children also gave their verbal consent to perform the measurements.

### 2.2. Data Collection

**Figure 2:**
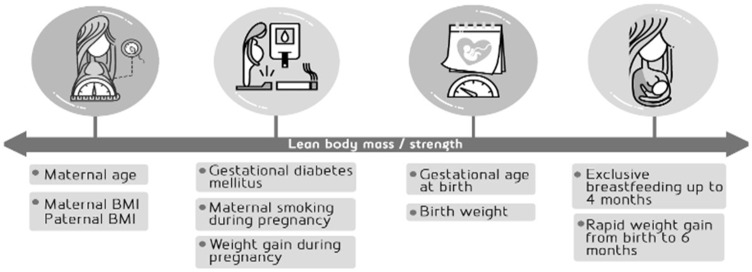
Early life factors as selected potential predictors of lean body mass/strength.

The methodology for collecting the early life factors following the timeline shown in Figure 2 and the body composition and limb strength results are presented below:

#### 2.2.1. Parental Factors

- Advanced Maternal Age: Obtained at birth from hospital records and categorized as <35 Years and ≥35 Years.
- Parents’ body mass index (BMI): Parents’ weight and height before pregnancy were obtained during a face-to-face interview. Subsequently, the BMI of each was calculated as the weight (kg) divided by height squared (m^2^) and classified according to the cut-off points of the World Health Organization (WHO) (normal weight 18.5–24.9 kg/m^2^; overweight 25– <30 kg/m^2^; and obese, ≥30 kg/m^2^).
- Gestational diabetes mellitus: Mothers were diagnosed with gestational diabetes mellitus when at least two of the following four plasma glucose levels (measured fasting, at 1, 2, and 3 h during the oral glucose tolerance test [33]) were met or exceeded: Fasting: 95 mg/dL (5.3 mmol/L); 1 h: 180 mg/dL (10.0 mmol/L); 2 h: 155 mg/dL (8.6 mmol/L); and 3 h: 140 mg/dL (7.8 mmol/L). Subsequently, they were classified as gestational diabetes mellitus or not.
- Maternal smoking during pregnancy: Mothers were considered smokers if they smoked during pregnancy, regardless of the number of cigarettes. Information was collected by interviewing the mothers before being discharged after delivery or from medical records. It was categorized as yes or no.
- Weight gain during pregnancy: was obtained from medical records and calculated as the difference between the maximum recorded weight during pregnancy and the self-reported weight before pregnancy. This value was used to classify weight gain during pregnancy as insufficient, adequate, or excessive according to the recommendations for healthy weight gain in pregnant women issued by the Institute of Medicine [34]. It uses the BMI before pregnancy as a reference (Pre-pregnancy BMI underweight (<18.5 kg/m^2^) = 12.5–18 kg; normal weight (18.5–24.9 kg/m^2^) = 11.5–16 kg; overweight (25–29.9 kg/m^2^) = 7–11.5 kg; and obese (≥30 kg/m^2^) = 5–9 kg).
- Gestational age at birth: was obtained through hospital records and categorized into preterm: <37 weeks and term: 37–42 weeks [35].
- Birth weight (BW)*:* measured just after infant delivery in the hospital and registered during the first visit by CALINA research staff. Categorized as low (<2.5 kg), normal (2.5− <4 kg), and high (≥4 kg) [36].
- Exclusive breastfeeding for at least four months: defined according to the WHO [37] as infant breast milk feeding without other solid or liquid supplements, including water.
- Rapid weight gain (RWG): according to the WHO child growth pattern tables [38], defined as an increase in weight for age z-score between BW and six months of age greater than 0.67 standard deviations (SD) [39].

#### 2.2.2. Body Composition (Outcome Variables) at Ages Six to Eight

- Bioimpedance analysis (BIA), weight and height): Bioelectrical impedance analysis and weight were measured with an electronic balance (TANITA BC 418 MA, Tanita Europe BV, Amsterdam, The Netherlands) with an accuracy of 100 g and a range of 0–200 kg, according to the manufacturer’s instructions.

Height was measured with a portable stadiometer (SECA^®^ 225, Hamburg, Germany) with a precision of 0.1 cm and a 70–220 cm range. Subsequently, BMI was calculated as the weight divided by the squared height (kg/m^2^). The specific z-scores for age and sex were calculated using the WHO AnthroPlus [38] software. Starting from the value of the FFM (kg), the fat-free mass index (FFMI) was estimated as the FFM in kilograms divided by the squared height in meters (FFM kg/m^2^). Fat mass index (FMI) was estimated as FM in kilograms divided by height squared in meters (FM kg/m^2^).

- Dual-energy X-ray absorptiometry (DXA): LBM (kg) in a whole-body examination was determined using DXA QDR-Explorer™ 4500 equipment (Hologic Inc., Bedford, Massachusetts, USA), following the manufacturer’s instructions [40]. The variation of our laboratory’s intra-measured LBM whole-body examination coefficient is 1.9% and has been previously described [41].

The present study used the value of lean soft tissue mass LSTM = LBM − bone mineral content. The total LSTM index (TLSTMI) was calculated from the values of LSTM (kg), as the TLSTM (kg) divided by the height squared in meters (LSTM kg/m^2^). Fat mass index (FMI) by DXA was estimated as FM in kilograms divided by height squared in meters (FM kg/m^2^).

- Peripheral quantitative computed tomography (pQCT): The Stratec XCT 2000 L (Stratec Medizintechnik, Pforzheim, Germany), explained elsewhere [42], was used to estimate the cross-sectional muscle area (MCSA) at 66% of the total length of the left tibia [42].

As previously reported, the intra-measured coefficient of variation for MCSA using pQCT was 1.69% [43].

The MCSA index (MCSAI) was calculated by dividing the MCSA by the squared height (MCSA/m^2^). Fat area index (FAI) was estimated as the fat area (FA) divided by height squared in meters (FA/m^2^).

A technician visually evaluated both the pQCT and DXA images to identify motion artifacts. Images showing movement were rescanned, when possible, or excluded from the analysis.

#### 2.2.3. Limb Strength (Outcome Variables) at Ages Six to Eight

- Handgrip strength test: According to the manufacturer’s instructions, handgrip strength was measured with a TKK-5401 digital grip dynamometer (Takei Scientific Instruments Co., Ltd., Niigata, Japan), with an accuracy of 0.1 kg.

The following gender-specific equations were used for proper adjustment to each child’s hand size [44]:

Boys: Y = X/4 + 0.44 cm

Girls: Y = 0.3X − 0.52 cm

Where Y = optimal grip and X = size of the open hand, measured from the tip of the thumb to the tip of the little finger.

Two attempts were made with each hand, with an interval of three minutes of rest between each of them. The final score was calculated as the mean of the best attempt obtained in kg by the left and right hands [45].

- Standing long jump test: From a stand-up position, with their feet slightly apart, the participants were instructed to push off with both feet using the arms’ impulse to complete the forward jump while avoiding stepping on the starting line. Results were measured from the heel drive line closest to the starting line. The highest value achieved in two attempts was recorded in cm [46].

#### 2.2.4. Potential Confounding Factors

- Parents ‘education: During the follow-up carried out in 2016–2017, both parents were asked to report their highest level of education attained (no studies; basic-primary studies; intermediate studies; higher education and university degrees). The results were subsequently coded according to the International Standard Classification of Education (ISCED-2011) [47] and reclassified as low (0–2), medium (3–4), and high (5–8) educational levels [48].
- Origin/ethnicity of the parents: The mothers were asked to report their children’s ethnicity/origin. The child was considered of immigrant origin if one or both parents had been born in a country other than Spain. Natives were those whose parents were born in Spain.

### 2.3. Statistical Analysis

Statistical analysis was performed using IBM SPSS Statistics^®^ software, version 25 (IBM Corp., Armonk, NY, USA). The distribution of the variables was verified using the Kolmogorov-Smirnov test. The variables following normal distribution were presented as means (M) ±, SD; in the case of variables with non-normal distribution, the median values and interquartile ranges (25th and 75th) were presented, whereas categorical variables were described as absolute frequencies. All analyses were performed separately for boys and girls. Differences between the studied variables were evaluated with the Student’s *t*-test or the Mann-Whitney U test, depending on the distribution of the variable. Maternal and paternal characteristics were compared using the Chi-squared test for categorical variables.

Multiple linear regression (Forced Entry) was used to study the association between early life factors (parental nutritional status, maternal smoking during pregnancy, gestational diabetes mellitus, gestational weight gain, gestational age, BW, breastfeeding practices, and RWG) and the body composition (FFMI, TLSTMI, MCSAI) and limb strength (handgrip strength, and standing long jump) outcomes. These associations were analyzed in individual regression models. Model 1 included each of the early life factors. Model 2 included Model 1 plus the children’s age in months. Model 3 included Model 2 plus the potential confounders for each early life risk factor considered relevant in the literature * plus the fat mass/fat area index. Model 4 included Model 2 plus the possible confounders for each early life risk factor found to be relevant in the literature * plus the weight of girls and boys. The *confounding factors for Models 3 and 4 were different for each dependent variable as follows (Table 1).

The assumptions of independence of errors were verified for all models using the Durbin-Watson test. A collinearity diagnosis was carried out through the variance inflation factor (VIF).

## 3. Results

The participants’ characteristics, including maternal and paternal characteristics, early life factors, anthropometric measurements, BIA, DXA, pQCT, and limb strength stratified by sex, are shown in Table 2. Boys showed higher levels of BMI z-score, FFM, FFMI, TLSTM, TLSTMI, MCSA, MCSAI, handgrip strength, and standing long jump results than girls (all *p* < 0.05).

Adjusted associations between early life factors and FFMI, TLSTMI, and MCSAI for both girls and boys are shown in Table 3. In girls, we observed a positive association between maternal smoking during pregnancy and FFMI (β = 0.163, *p* = 0.040) and TLSTMI (β = 0.238, *p* = 0.003) for Model 1. In the case of the association between maternal smoking during pregnancy and FFMI, it disappeared in the other models. In Model 2, adding the girls’ age in months, an association was found between maternal smoking during pregnancy and TLSTMI (β = 0.226, *p* = 0.003). When maternal education, origin/ethnicity, and FMI were added to create Model 3, significant associations were found between maternal smoking during pregnancy and TLSTMI (β = 0.188, *p* = 0.009) (Table 3). Finally, after adjusting for Model 2 + child weight (Model 4), significant associations were found between maternal smoking during pregnancy and TLSTMI (β = 0.191, *p* = 0.002) (Table 3).

On the other hand, we observed a positive association between gestational age and FFMI (β = 0.162, *p* = 0.047) and TLSTMI (β = 0.230 *p* = 0.004) in Model 1, Model 2 (FFMI β = 0.176, *p* = 0.029; TLSTMI β = 0.244, *p* = 0.002) and Model 3 (FFMI β = 0.133, *p* = 0.030; TLSTMI β = 0.189, *p* = 0.006). In Model 4, an association was only observed between gestational age and TLSTMI (β = 0.126, *p* = 0.031).

Also, we observed a positive association between BW and FFMI (β = 0.170, *p* = 0.022) in Model 1, Model 2 (β = 0.172, *p* = 0.019) and Model 3 (β = 0.143, *p* = 0.009). Regarding the TLSTMI, we observed a positive association in all models (Models 1 and 2 β = 0.276, *p* = 0.000; Model 3 β = 0.304, *p* = 0.000, and Model 4 β = 0.160, *p* = 0.010).

For its part, in girls, we observed a positive association between handgrip strength and BW in Model 1 (β = 0.193, *p* = 0.010), Model 2 (β = 0.192, *p* = 0.010), and Model 3 (β = 0.200, *p* = 0.008) (Table 4). Regarding the standing long jump, we observed a negative association with maternal BMI (β = −0.169, *p* = 0.038) in Model 1 and Model 2 (β = −0.167, *p* = 0.041) (Table 4).

The positive association is reported under Model 1 between maternal BMI and FFMI by BIA (β = 0.175, *p* = 0.023) in boys, an association maintained in Model 2 but not in Models 3 and 4 (Table 2). Regarding paternal BMI, a positive association with TLSTMI by DXA was found in Model 1 (β = 0.185, *p* 0.019), Model 2 (β = 0.217, *p* = 0.006) and disappeared in Models 3 and 4. For BW, we observed a positive association with TLSTMI (β = 0.226, *p* = 0.001) in Model 1, Model 2 and Model 3 (β = 0.221, *p* = 0.001; β = 0.182, *p* = 0.001, respectively).

Finally, for boys, associations were found between RWG and FFMI in Models 1 and 2 (β = 0.182, *p* = 0.041; β = 0.180, *p* = 0.043, respectively). For TLSTMI, no associations were found in Model 1 and Model 2 but a positive association was observed in Model 3 (β = 0.700, *p* = 0.017).

Likewise, the association between handgrip and BW in boys showed a similar trend to that of girls (Model 1 β = 0.279; Model 2 β = 0.272, and Model 3 β = 0.253, *p* < 0.001 in all cases). Regarding the standing long jump, for boys, we observed a negative association and RWG in Model 1(β = −0.308, *p* = 0.004) and Model 2 (β = −0.309, *p* = 0.004); but this disappeared in Models 3 and 4 (Table 4).

## 4. Discussion

Our findings reported that maternal smoking exposure in utero in girls is associated with FFM/LSTM measured with BIA and DXA. Leary et al. (β = 0.39, *p* <0.001) [49] and da Silva et al. (β = 0.33, *p* < 0.001) [50] also observed that lean mass was higher among subjects whose mothers smoked during pregnancy. The association of maternal smoking and FFM/LSTM may be due to the association of maternal smoking during pregnancy with an increased risk of obesity in childhood [51]. Quite possibly the associations involving lean mass simply reflect those of fat mass, as larger children will have more fat than lean mass [50]. However, we could not confirm this hypothesis by controlling for the FMI. Nonetheless, we observed that greater lean mass was not associated with greater limb strength at the age of six to eight years.

Another mechanism of association between maternal smoking and higher lean mass can be found in current evidence suggesting that although smoking affects muscle protein synthesis [lower mixed muscle protein fractional synthesis rate (FSR) and increased expression of genes involved in muscle mass-myostatin regulation and muscle atrophy F-box (MAFBx)] [52], smoking cessation is associated with increased muscle mass and strength. Thus, even though the mothers of the children participating in the CALINA study continued to smoke during pregnancy, they likely reduced their consumption, favoring the synthesis of muscle proteins in the fetus [53]. However, this hypothesis could not be confirmed, given that we did not have data on any change in smoking pattern/frequency during pregnancy.

In contrast, other studies have found an association between maternal smoking and decreased FFM in the neonatal period [54,55]. This result may be due to the effects of nicotine on BW, including hypoxia secondary to vasoconstriction, impaired placental function, impaired protein synthesis, and accumulation of lipids in cells. It may also be due to folic acid deficiency in the mother (necessary nutrient for protein synthesis), which is common during pregnancy and more pronounced in smokers [56].

Concerning gestational age, a study performed with preterm infants found that the % FFM was significantly lower in extremely preterm infants (born <28 weeks gestational age) than in very preterm infants (born ≥28 weeks gestational age) [57]. These findings align with our results, showing a positive association between gestational age and the fat-free/lean soft tissue mass in girls. This result could indicate that the last trimester of pregnancy is potentially a critical period for the programming of FFM/LSTM, as pointed out previously [58]. Furthermore, gestational age contributes significantly to BW [59], a factor that, as mentioned, can eventually contribute to LBM.

Consistent with previous studies, we also observed a relationship between BW and LBM. In a study by Beltrand et al. [60], where 235 low-risk pregnancies were included and newborns were evaluated at birth, it was observed that those with the lowest fetal growth rate tertile showed severe fetal growth restriction. This condition was strongly associated with a reduction in LBM in both genders (*p* <0.001). Similarly, a longitudinal study including 39 ex-babies (22 female, 17 male) with extremely low BW found a positive correlation between the BW SD score and LBM in 9.5-year-olds [61]. In another study, Ylihärsilä et al. [62] showed that an increase of one kg in BW increased lean mass in male adults by 4.1 kg and 2.9 kg in women. This association persisted significantly after adjustment for age, adult body size, physical activity, smoking, social class, and maternal height.

The mechanisms explaining the association of BW and low LBM could be fetal nutrition, hormonal status, socioeconomic status, and postnatal factors. One mechanism, for instance, could be suboptimal nutrition in the uterus, predisposing the individual to fetal hypoglycemia, limiting insulin secretion, and, consequently, increasing protein breakdown and decreasing its accumulation. Impaired nutrition could also reduce the levels of insulin-like growth factor 1 (IGF-1) [16], which plays a crucial role in fetal growth, more specifically in the growth of LBM, organs, and the skeleton [63]. Regarding socioeconomic status, some studies have shown that lower socioeconomic status (β = 0.17; *p* <0.05) and BW (β = −0.11, *p* <0.05) was associated with lower muscle mass (%). This link between socioeconomic status and muscle mass may be related to the quality of life and having access to various types of physical activities and healthy foods [64].

It is unclear whether the association between BW and LBM may also be due to postnatal factors. Low BW is often associated with postnatal catch-up growth. Our results indicate a positive association between RWG, LSTM, and FFM in girls. Similar results were found by Euser et al. [65] in young adults for both genders. However, other studies have found a postnatal catch-up growth of adipose tissue that exceeds that of LBM [24].

Similar to our results involving the parents’ nutritional status, other studies have found that the mother’s BMI was positively associated with both their male and female children’s LMI z-scores. However, the father’s BMI showed positive associations only with the male’s LMI z-scores [15]. Multiple routes may support these associations through which each parent can affect their offspring’s phenotype [66]. From an evolutionary perspective, the parent’s influence on their children’s body composition is presumably derived from the different strategies used to maximize their reproductive fitness. However, behavioral mechanisms can also be an influence; for instance, mothers tend to be more involved in all aspects of child-rearing, influencing children’s lifestyles and eating habits [15].

Finally, sexual dysmorphism could also explain our study’s findings on the associations between different early life factors and FFM/LSTM in girls and boys. This has already been described to influence child growth trajectories and body composition parameters such as the FFM [67,68].

Our study found an association between BW and handgrip, coinciding with the results found by Ahlqvist et al. [69] in a cohort of 144,369 young men born at term. This result may be because the number of muscle fibers in mammals is determined at or shortly after birth and influenced by nutritional status during critical periods of development [70]. Although postnatal muscle growth is due to muscle fiber growth [71], those with fewer fibers will have a future disadvantage in terms of muscle growth.

We also observed a negative association between maternal BMI and their daughters’ standing long jump test, an association previously reported in other studies [72,73]. This result could be because an obesogenic environment during pregnancy is associated with increased leptin levels, seemingly affecting fetal muscle growth. In addition, animal model studies have found a reduced expression of glucose transporter type 4 (GLUT4) and myogenic differentiation 1 genes in the offspring of obese mothers, which could also be a mechanism that alters muscle function [74]. The negative association between lower extremity strength and maternal BMI may be due to the girls’ BMI on this test, which is considered weight-dependent and requires propulsion or elevation of the body. Therefore, girls with higher weight may not perform as well on this test.

Our results, like previous studies, show a negative association between the RWG and the standing long jump test of the boys [75]. This association may be because RWG is also associated with an increased risk of subsequent overweight and obesity [76], which may also advance the rebound time of adiposity [75]. Thus, considering that this test is dependent on the participant’s weight, this may affect the children’s performance.

This study was carried out with Spanish children in a particular age group (ages six to eight). Thus, the outcomes are limited to this set of participants. Further studies should consider different age and ethnic groups. Furthermore, the observed associations cannot be interpreted as causal relationships. Another limitation is the reliance on parental self-report measures, such as parental weight and height, smoking during pregnancy, and educational level. Finally, it is possible that some associations did not reach statistical significance, perhaps due to the relatively small sample size. However, our sample was larger than those of some studies whose results have been contrasted in some cases [54,57,60,61].

Despite these limitations, our study has some noteworthy strengths. To our knowledge, this is the first study that researches early life factors and their effect as a predictor of fat-free/lean soft tissue mass using a Spanish cohort, followed up at ages six to eight. Another strength is the prospective collection of data on a wide range of risk factors extending from pregnancy to infancy and their adjustment to different confounders. Lastly, the two techniques used to determine FFM/LSTM, namely BIA and DXA, are the most accessible methods for evaluating the general public and the accepted gold standard method for evaluating LBM, respectively.

## 5. Conclusions

Our findings suggest that early life programming has an important role in determining lean body mass. However, more future studies are needed to better clarify the relationships between early life factors, fat-free mass, and lean soft tissue mass in children and later stages in life, taking into account other factors such as genetic factors or the abuse of toxic substances during pregnancy.

## Figures and Tables

**Table 1:** The Confounding factors and adjustment.

Confounding Factors	Adjustment
Maternal age	Adjustment by maternal education.
Maternal BMI	Adjustment by maternal education, origin/ethnicity, and maternal smoking during pregnancy.
Paternal BMI	Adjustment by paternal education and origin/ethnicity.
Gestational diabetes mellitus	Adjustment by maternal age, maternal BMI, and maternal smoking during pregnancy
Maternal smoking during pregnancy	Adjustment by maternal education, and origin/ethnicity.
Weight gain during pregnancy	Adjustment by maternal BMI, maternal smoking during pregnancy, maternal education, and gestational age.
Gestational age	Adjustment by maternal smoking during pregnancy and maternal education.
Birth weight	Adjustment by maternal smoking during pregnancy and maternal BMI
Exclusive breastfeeding	Adjustment by origin/ethnicity, maternal education, maternal BMI, and maternal smoking during pregnancy.
Rapid weight gain	Adjustment by BW, maternal BMI, paternal BMI, maternal education, and origin/ethnicity.

**Table 2:** Descriptive characteristics of the study population by sex at last follow-up (six to eight years old) (*n* = 416).

Characteristics	All *n* = 416	Girls *n* = 197	Boys *n* = 219	*p*-Value
Age (months)	91 (88–93)	91(87–94)	91(88–93)	0.41
**Maternal and paternal characteristics**				
Maternal age at bith	34 (31–36)	34 (31–36)	33 (30–36)	0.09
<35 years	295 (70.9%)	135 (45.8%)	160 (54.2%)	
≥35 years	121 (29.1%)	62 (51.2%)	59 (48.8%)	
Maternal education				
Missing	76 (18.3%)	35 (46.1%)	41 (53.9%)	0.26
Low	31 (7.5%)	12 (38.7%)	19 (61.3%)	
Medium	143 (34.3%)	76 (53.1%)	67 (46.9%)	
High	166 (39.9%)	74 (44.6%)	92 (55.4%)	
Paternal education				
Missing	85 (20.4%)	42 (49.4%)	43 (50.6%)	0.45
Low	38 (9.1%)	22 (57.9%)	16 (42.1%)	
Medium	188 (45.2%)	88 (46.8%)	100 (53.2%)	
High	105 (25.3%)	45 (42.9%)	60 (57.1)	
Parental origin/ethnicity				
Spanish	357 (85.8%)	163 (45.7%)	194 (54.3%)	0.07
Other	59 (14.2%)	34 (57.6%)	25 (42.4%)	
**Early factors**				
Maternal BMI	22.7 (20.7–25.7)	22.3 (20.5–25.5)	23.0 (21.0–25.9)	0.20
Normal weight	296 (71.2%)	143 (48.3%)	153 (51.7%)	
Overweight	82 (19.7%)	37 (45.1%)	45 (54.9%)	
Obese	38 (9.1%)	17 (44.7%)	21 (55.3%)	
Paternal BMI	25.5 (23.8–27.5)	25.3 (23.5–27.2)	25.6 (24.2–27.8)	0.01 *
Normal weight	177 (42.5%)	93 (52.5%)	84 (47.5%)	
Overweight	194 (46.7%)	86 (44.3%)	108 (55.7%)	
Obese	45 (10.8%)	18 (40%)	27 (60%)	
Gestational diabetes mellitus				
Yes	39 (9.4%)	11 (28.2%)	28 (71.8%)	0.01 *
No	377 (90.6%)	186 (49.3%)	191 (50.7%)	
Maternal smoking during pregnancy				
Yes	66 (15.9%)	29 (43.9%)	37 (56.1%)	0.53
No	350 (84.1%)	168 (48%)	182 (52%)	
Weight gain during pregnancy	11 (9.0–14)	11 (8.7–14)	11 (9.0–14)	0.26
Insufficient	164 (39.4%)	84 (51.2%)	80 (48.8%)	
Adequate	160 (38.5%)	77 (48.1%)	83 (51.9%)	
Excessive	92 (22.1%)	36 (39.1%)	56 (60.9%)	
Gestational age	39 (38–40)	39 (38–40)	39 (38–40)	0.07
<37 weeks	64 (15.4%)	21 (32.8%)	43 (67.2%)	
37– 42 weeks	352 (84.6%)	176 (50%	176 (50%)	
Birth weight	3208 ± 505	3157 ± 458	3254 ± 540	0.05
<2.5 kg	0 (0.0%)	0 (0.0%)	0 (0.0%)	
2.5– <4 kg	373 (89.7%)	182 (48.8%)	191 (51.2%)	
≥4 kg	43 (10.3%)	15 (34.9%)	28 (65.1%)	
Exclusive breastfeeding at month four				
Yes	142 (34.1%)	72 (50.7%)	70 (49.3%)	0.42
No	274 (65.9%)	125 (45.6%)	149 (54.4%)	
Rapid infant weight gain	0.1 ± 1.1	0.6 ± 0.98	0.2 ± 1.2	0.45
Yes	121 (29.1%)	49 (40.5%)	72 (59.5%)	
No	295 (70.9%)	148 (50.2%)	147 (49.8%)	
**Children’s anthropometric parameters**				
Height (cm)	126.1 ± 5.9	125.6 ± 5.7	125.5 ± 6.0	0.12
Weight (kg) ^a^	26.4 (23.6–30.0)	26.2 (23.7–29.9)	26.5 (23.5–30.2)	0.59
BMI (kg/m^2^) ^a^	16.6 (15.4–18.3)	16.7 (15.4–18.3)	16.5(15.4–18.4)	0.74
BMI z-score ^†^	0.8 ± 1.2	0.7 ± 1.1	0.8 ± 1.3	<0.01 *
**BIA**				
FFM (kg) ^a^	20.5 (18.7–22.6)	19.9 (18.5–21.8)	20.8(18.9–23.3)	<0.01 *
FFMI (kg/m^2^)	13.0 (12.2–13.8)	12.7 (12.1–13.4)	13.1 (12.4–14.1)	<0.01 *
FMI (kg/m^2^)	3.6 (3.0–4.7)	3.8 (3.4–4.9)	3.4 (2.9–4.4)	<0.01 *
**DXA**				
TLSTM (kg) ^a^	19.0 (17.4–20.9)	18.5 (17.0–19.6)	19.7 (18.0–22.0)	<0.01 *
TLSTMI (kg/m^2^)	12.0 (11.4–12.8)	11.6 (11.1–12.3)	12.4 (11.8–13.2)	<0.01 *
FMI (kg/m^2^)	4.4 (3.6–5.9)	4.8 (4.1–6.2)	4.0 (3.3–5.4)	<0.01 *
**pQCT**				
Tibia length (mm)	274 ± 18	276 ± 17	273 ± 19	0.17
MCSA (mm^2^)	3195.2 (2931.0–3591.0)	2585.8 (2547.8–2619.1)	2794.2 (2750.5–2846.0)	<0.01 *
MCSAI (mm^2^/m^2^)	2029.4 (1893.8–2225.9)	1976.8 (1823.4–2139.0)	2108.9 (1964.7–2295.7)	<0.01 *
FA (mm^2^)	1639.3 (1308.5–2099.5)	1782.0 (1413.5–2149.8)	1527.5 (1222.0–2039.0)	<0.01 *
FAI (mm^2^/m^2^)	1034.1 (939.3–1289.9)	1124.2 (927.5–1322.6)	957.9 (764.0–1226.3)	<0.01 *
**Limb strength**				
Handgrip strength (kg)	10.5 ± 2.2	10.2 ± 2.1	10.8 ± 2.3	<0.01 *
Standing long jump (cm)	102.8 ± 17.8	98.9 ± 17.0	107.6 ± 17.7	<0.01 *

Abbreviations: Body mass index (BMI); Bioelectrical impedance (BIA); Fat-free mass (FFM); Fat-free mass index (FFMI); Fat mass index (FMI); Dual-energy X-ray absorptiometry (DXA); Total lean soft tissue mass (TLSTM); Total lean soft tissue mass index (TLSTMI); quantitative peripheral computed tomography (pQCT); Muscle cross-sectional area (MCSA); Fat area (FA) and Fat area index (FAI). ^†^ BMI z-scores were calculated according to the World Health Organization (WHO). Mean ± SD (Student *t*-test) represents the normally distributed variables. ^a^ Non-normally distributed variables are shown as median and interquartile intervals (25th and 75th, U Mann–Whitney). Statistical analyses were undertaken using Student’s *t*-tests (for continuous variables) and chi-square tests (for categorical variables). * Significant differences by gender. Significance was set at a level of 0.05.

**Table 3:** Associations between early life factors and FFMI, TLSTMI and MCSAI in girls and boys at six years old.

Predictors Early Life Risk Factors	Girls (*n* = 197)																							
	FFMI by BIA								TLSTMI by DXA								MCSAI by pQCT							
	Model 1		Model 2		Model 3		Model 4		Model 1		Model 2		Model 3		Model 4		Model 1		Model 2		Model 3		Model 4	
	β	*p*	β	*p*	β	*p*	β	*p*	β	*p*	β	*p*	β	*p*	β	*p*	β	*p*	β	*p*	β	*p*	β	*p*
Maternal age ^a^	−0.165	0.076	−0.176	0.054	−0.109	−0.113	−0.129	0.039	−0.067	0.476	−0.082	0.374	−0.019	0.808	−0.060	0.396	0.073	0.450	0.072	0.462	0.085	0.367	0.077	0.399
Maternal BMI ^b^	0.040	0.624	0.051	0.525	−0.016	0.788	−0.051	0.353	0.003	0.975	0.015	0.852	−0.070	0.321	−0.079	0.191	0.146	0.079	0.146	0.080	0.103	0.209	0.098	0.221
Paternal BMI ^c^	0.149	0.074	0.153	0.062	−0.013	0.849	−0.046	0.461	0.095	0.253	0.100	0.225	−0.035	0.647	−0.084	0.214	−0.003	0.969	−0.004	0.963	−0.064	0.446	−0.134	0.112
Gestational diabetes mellitus ^d^	−0.083	0.334	−0.067	0.430	−0.054	0.385	−0.025	0.668	−0.045	0.599	−0.029	0.732	−0.018	0.813	0.015	0.819	−0.019	0.833	−0.019	0.829	−0.031	0.717	−0.009	0.918
Maternal smoking during pregnancy ^e^	0.163	0.040	0.151	0.054	0.075	0.228	0.095	0.092	0.238	0.003	0.226	0.003	0.188	0.009	0.191	0.002	0.118	0.150	0.117	0.153	0.098	0.240	0.102	0.207
Weight gain during pregnancy ^f^	0.008	0.922	0.002	0.984	0.016	0.799	−0.023	0.684	0.056	0.501	0.049	0.545	0.093	0.183	0.017	0.781	0.049	0.569	0.049	0.570	0.114	0.169	0.081	0.326
Gestational age ^g^	0.162	0.047	0.176	0.029	0.133	0.030	0.081	0.136	0.230	0.004	0.244	0.002	0.189	0.006	0.126	0.031	0.097	0.248	0.100	0.240	0.055	0.486	0.058	0.458
Birth weight ^h^	0.170	0.022	0.172	0.019	0.143	0.009	0.064	0.220	0.276	0.000	0.276	0.000	0.304	0.000	0.160	0.010	0.121	0.116	0.121	0.117	0.150	0.043	0.106	0.163
Exclusive breastfeeding ^i^	0.082	0.366	0.077	0.390	−0.016	0.816	−0.024	0.667	0.110	0.221	0.099	0.267	0.034	0.670	0.011	0.873	0.031	0.735	0.037	0.696	−0.022	0.811	−0.049	0.577
Rapid infant weight gain ^j^	0.048	0.612	0.039	0.685	0.002	0.984	−0.049	0.486	0.017	0.861	0.010	0.917	0.118	0.227	0.023	0.802	0.005	0.962	0.008	0.933	0.179	0.103	0.130	0.232
	**Boys (*n* = 219)**																							
Maternal age ^a^	0.052	0.576	0.060	0.521	0.029	0.730	0.026	0.744	−0.067	0.471	−0.080	0.391	−0.082	0.221	−0.092	0.119	−0.037	0.694	−0.037	0.697	−0.051	0.558	−0.061	0.482
Maternal BMI ^b^	0.175	0.023	0.175	0.023	0.074	0.269	0.075	0.230	0.088	0.257	0.098	0.203	−0.030	0.615	−0.021	0.687	0.103	0.194	0.104	0.187	0.003	0.964	0.008	0.914
Paternal BMI ^c^	0.101	0.203	0.103	0.201	−0.002	0.982	−0.049	0.470	0.185	0.019	0.217	0.006	0.105	0.098	0.033	0.542	0.116	0.152	0.127	0.122	0.053	0.500	0.030	0.710
Gestational diabetes mellitus ^d^	−0.030	0.719	−0.031	0.716	−0.019	0.787	0.029	0.664	0.045	0.600	0.047	0.580	0.114	0.077	0.148	0.009	0.115	0.190	0.115	0.192	0.140	0.097	0.157	0.062
Maternal smoking during pregnancy ^e^	−0.026	0.734	−0.026	0.734	−0.004	0.951	0.003	0.961	0.045	0.559	0.041	0.590	0.059	0.321	0.072	0.158	0.004	0.962	0.002	0.982	0.010	0.890	0.012	0.877
Weight gain during pregnancy ^f^	−0.037	0.637	−0.043	0.585	0.014	0.799	0.046	0.325	0.011	0.892	0.003	0.971	0.039	0.514	0.077	0.129	−0.025	0.756	−0.027	0.743	−0.005	0.948	0.003	0.967
Gestational age ^g^	0.031	0.696	0.043	0.583	0.048	0.360	−0.003	0.954	0.099	0.206	0.119	0.128	0.133	0.024	0.088	0.082	−0.083	0.299	−0.082	0.310	−0.047	0.539	−0.055	0.466
Birth weight ^h^	0.068	0.325	0.068	0.329	0.017	0.763	−0.090	0.094	0.226	0.001	0.221	0.001	0.182	0.001	0.073	0.126	−0.048	0.503	−0.049	0.495	−0.085	0.211	−0.118	0.086
Exclusive breastfeeding ^i^	0.009	0.913	0.010	0.912	−0.027	0.718	−0.073	0.299	0.053	0.541	0.053	0.533	0.026	0.681	−0.027	0.618	0.059	0.502	0.059	0.506	0.015	0.853	0.007	0.933
Rapid infant weight gain ^j^	0.182	0.041	0.180	0.043	0.111	0.224	−0.016	0.860	0.085	0.347	0.089	0.320	0.170	0.017	0.038	0.566	0.116	0.206	0.116	0.207	0.064	0.497	0.035	0.720

β: standardized regression coefficient. Abbreviations: Body mass index (BMI); Bioimpedance analysis (BIA); Dual-energy X-ray absorptiometry (DXA); Fat-free mass index (FFMI); Muscle cross-sectional area index (MCSAI); Peripheral quantitative computed tomography (pQCT); Total lean soft tissue mass index (TLSTMI). *Model 1* early life factors (basic model without adjustments). *Model 2 Model 1* + children’s age in months. *Model 3 Model 2* + possible confounders for each early life factor that have been found to be relevant in the literature as covariates* + fat mass/fat area index. *Model 4 Model 2* + possible confounders for each early life risk factor that have been found to be relevant in the literature as covariates* + weight of girls and boys. *Confounding factors. ^a^ Maternal age: Maternal education. ^b^ Maternal BMI: Maternal education, origin/ethnicity, and maternal smoking during pregnancy. ^c^ Paternal BMI: Paternal education and origin/ethnicity. ^d^ Gestational diabetes mellitus: Maternal age, maternal BMI, and maternal smoking during pregnancy. ^e^ Maternal smoking during pregnancy: Maternal education and origin/ethnicity. ^f^ Weight gain during pregnancy: Maternal BMI, maternal smoking during pregnancy, maternal education, and gestational age. ^g^ Gestational age: Maternal smoking during pregnancy and maternal education. ^h^ Birth weight: Maternal smoking during pregnancy and maternal BMI. ^i^ Exclusive breastfeeding: Origin/ethnicity, maternal education, maternal BMI, and maternal smoking during pregnancy. ^j^ Rapid infant weight gain: Birth weight, breastfeeding, maternal BMI, paternal BMI, maternal education, and origin/ethnicity. Significance was set at a 0.05 level.

**Table 4:** Associations between early life factors and handgrip strength and standing long jump in girls and boys at six years old.

Predictors Early Life Risk Factors	Girls (*n* = 197)															
	Handgrip Strength								Standing Long Jump							
	Model 1		Model 2		Model 3		Model 4		Model 1		Model 2		Model 3		Model 4	
	β	*p*	β	*p*	β	*p*	β	*p*	β	*p*	β	*p*	β	*p*	β	*p*
Maternal age ^a^	0.026	0.784	0.015	0.873	0.016	0.867	−0.042	0.597	0.084	0.377	0.077	0.413	0.010	0.907	−0.022	0.792
Maternal BMI ^b^	−0.029	0.724	−0.017	0.833	−0.010	0.905	−0.021	0.766	−0.169	0.038	−0.167	0.041	−0.115	0.145	−0.119	0.113
Paternal BMI ^c^	−0.087	0.298	−0.080	0.334	−0.064	0.477	−0.123	0.100	−0.110	0.193	−0.109	0.197	0.012	0.891	−0.018	0.821
Gestational diabetes mellitus ^d^	−0.075	0.390	−0.061	0.483	−0.034	0.697	0.012	0.864	0.007	0.933	0.014	0.875	−0.003	0.974	0.016	0.847
Maternal smoking during pregnancy ^e^	0.084	0.298	0.073	0.356	0.074	0.395	0.073	0.303	−0.011	0.896	−0.013	0.869	0.049	0.540	0.051	0.512
Weight gain during pregnancy ^f^	0.091	0.274	0.088	0.282	0.105	0.239	0.011	0.880	0.085	0.307	0.085	0.308	−0.017	0.835	−0.065	0.415
Gestational age ^g^	0.025	0.761	0.037	0.650	0.027	0.750	−0.055	0.443	0.107	0.196	0.109	0.189	0.096	0.227	0.059	0.445
Birth weight ^h^	0.193	0.010	0.192	0.010	0.200	0.008	−0.038	0.576	0.113	0.124	0.117	0.124	0.104	0.140	0.046	0.549
Exclusive breastfeeding ^i^	0.153	0.090	0.145	0.108	0.113	0.239	0.092	0.266	0.027	0.765	0.024	0.794	0.058	0.485	0.048	0.546
Rapid infant weight gain ^j^	0.016	0.870	0.004	0.964	0.205	0.076	0.078	0.448	−0.131	0.174	−0.134	0.166	−0.001	0.994	−0.090	0.360
	Boys (*n* = 219)															
Maternal age ^a^	0.041	0.663	0.025	0.789	0.012	0.894	−0.003	.970	−0.173	0.121	−0.169	0.134	−0.122	0.293	−0.125	0.283
Maternal BMI ^b^	−0.090	0.250	−0.079	0.305	−0.140	0.068	−0.130	0.048	−0.079	0.396	−0.083	0.379	−0.007	0.944	−0.007	0.945
Paternal BMI ^c^	−0.027	0.739	0.007	0.928	−0.034	0.687	−0.136	0.058	0.041	0.670	0.038	0.700	0.163	0.121	0.121	0.255
Gestational diabetes mellitus ^d^	−0.141	0.101	−0.138	0.102	−0.109	0.190	−0.058	.407	0.030	0.777	0.030	0.779	0.062	0.550	0.092	0.374
Maternal smoking during pregnancy ^e^	−0.016	0.834	−0.019	0.799	−0.022	0.722	0.033	.966	−0.036	0.693	−0.037	0.690	−0.021	0.815	−0.017	0.847
Weight gain during pregnancy ^f^	−0.083	0.295	−0.095	0.227	−0.099	0.206	−0.060	.375	0.008	0.932	0.009	0.923	0.003	0.977	0.015	0.874
Gestational age ^g^	0.065	0.412	0.088	0.258	0.098	0.203	0.029	.658	0.177	0.058	0.176	0.063	0.203	0.026	0.178	0.054
Birth weight ^h^	0.279	0.000	0.272	0.000	0.253	0.000	0.123	0.034	0.059	0.482	0.058	0.494	0.070	0.375	0.010	0.903
Exclusive breastfeeding ^i^	0.090	0.304	0.089	0.299	0.082	0.330	0.012	.866	−0.152	0.161	−0.152	0.163	−0.105	0.315	−0.129	0.214
Rapid infant weight gain ^j^	0.002	0.979	0.006	0.944	0.190	0.049	0.006	.949	−0.308	0.004	−0.309	0.004	−0.187	0.134	−0.212	0.091

β: standardized regression coefficient. Abbreviations: BMI = Body − mass index. *Model 1* early life risk factors (basic model without adjustments). *Model 2 Model 1* + children’s age in months. *Model 3 Model 2* + possible confounders for each early life factor found to be relevant in the literature as covariates* + fat mass/fat area index. *Model 4 Model 2* + possible confounders for each early life risk factor found to be relevant in the literature as covariates* + weight of girls and boys. *Confounding factors. ^a^ Maternal age: Adjustment by maternal education. ^b^ Maternal BMI: Adjustment by maternal education, origin/ethnicity, and maternal smoking during pregnancy. ^c^ Paternal BMI: Adjustment by paternal education and origin/ethnicity. ^d^ Gestational diabetes mellitus: Adjustment by maternal age, maternal BMI, and maternal smoking during pregnancy. ^e^ Maternal smoking during pregnancy: Adjustment by maternal education and origin/ethnicity. ^f^ Weight gain during pregnancy: Adjustment by maternal BMI, maternal smoking during pregnancy, maternal education, and gestational age. ^g^ Gestational age: Adjustment by maternal smoking during pregnancy and maternal education. ^h^ Birth weight: Adjustment by maternal smoking during pregnancy and maternal BMI. ^i^ Exclusive breastfeeding: Adjustment by origin/ethnicity, maternal education, maternal BMI, and maternal smoking during pregnancy. ^j^ Rapid infant weight gain: Adjustment by birth weight, breastfeeding, maternal BMI, paternal BMI, maternal education, and origin/ethnicity. Significance level was set at 0.05.

## Data Availability

The data presented in this study are available on request from the corresponding author. The data are not publicly available due to data protection issues.
